# System in the Medical Student's Career

**Published:** 1909-09-04

**Authors:** 


### The Medical Student and his Work.

### SYSTEM IN THE MEDICAL STUDENT'S CAREER.

With the possible exception of those medical students whose fathers are members of the profession, there are probably few indeed who at the commencement of their studies have any correct perspective of the relative importance of the various branches of study outlined for them in the medical curriculum, or of the relative value to them in after years of the various parts of their medical education, or even of the different kinds of examination they are setting out to pass.

Those students of medicine who are sufficiently alive to the necessity of taking thought what their programme for the next five years or so is to be, and have taken the trouble to get the advice of their Dean or other constituted authority, are generally impressed with the usefulness of a degree in medicine as opposed to diplomas alone, and they direct their studies from the outset with the aim and object of acquiring that particular degree which chance or circumstances happen to render most accessible or most coveted. Others, less willing to take advice, less disposed towards the extra exertion commonly entailed in the preliminary examinations, or less able to face the additional financial burden, set themselves a lower standard from the first, and this group includes a very considerable proportion of the whole number. It is one of the commonplaces of medical school gossip how often the latter come to regret eventually the lost opportunities of their early days, and how hard those who do make up their minds to retrieve the position must work to regain the status of their more far-seeing contemporaries.

On the other hand, when all is said and done, it is also to be admitted that many, very many, of those who begin with the idea of taking a degree in medicine never complete the necessary exercises, and remain content with the diplomas, which are after all a complete and satisfactory guarantee of professional efficiency, but one with a rather lower market value in the eyes of the non-medical public. And of those who thus begin to work for a degree, but never obtain it, there are many who have wasted in fees and in extra study for the first and second professional examinations time and money in very fairly considerable quantities. So that in commending indiscriminately to medical students that desire for the best which is certainly preferable to a dogged contentment with the second best, it must not be overlooked that there are those of whom it is safe to prophesy that too ambitious a scheme is likely to be a waste of energy.

Taking this into account, and taking into account also a plain truth which is seldom appreciated by students, that there is really no examination in the whole range of degrees or diplomas which is outside the capacity of a man of strictly average ability *if he will work*, it remains to be said that the intelligent student who means to get the best out of his studies and to fit himself for a successful career when student days are done should start with at least a rough scheme of how long his curriculum is to take him and what it is to comprise. He should have clearly fixed in his mind how many years he can spare for his anatomy and physiology, and what examinations he means to pass in those subjects. He should, if he means to enter ultimately for the Fellowship of the Royal College of Surgeons, allow himself time not only for the Intermediate examination of the particular university or corporation from which he expects finally to acquire his qualification, but also for the primary Fellowship, as it is commonly called. To go back from his ward work after qualification, and to pick up afresh the ravelled threads of anatomy and physiology is a task which is undertaken every year by scores of men who curse the blindness which caused them to neglect the said "primary" after passing their Intermediate examination.

Leaving out of account those men who wander off from the main high road to medical practice to cultivate the by-paths of zoology, physiology, and the other sciences included in the first half of the medical student's training, the rule should be faithfully kept of geting rid of one sub-division of the medical course before attempting to tackle the next. The student who still has any examination to pass in chemistry, physics, or zoology should on no account whatever be tempted to commence the study of anatomy or physiology; and similarly he who has still any professional test of the latter subjects to satisfy should rigidly exclude bacteriology, pathology, or clinical work of any kind until he has overpast it. It may seem superfluous to insist on such an elementary principle of doing with all one's might whatsoever the hand findeth to do; but experience has shown that there is a certain proportion of men who cannot resist the temptation to dabble in study not strictly germane at the moment to the advancement of their careers, and more than one instance could be quoted of men who have failed again and again at anatomy because of an irresistible inclination to prefer the study of pathology or of clinical surgery.

The prudent man, then, is he who sketches out roughly for himself the qualifications he wishes to take—erring, if at all, on the ambitious side rather than the self-distrustful—and the length of time he proposes, examiners willing, to devote to the various compartments of his progress thereunto. Upon the great majority, those who intend to make the daily practice of medicine and surgery their means of livelihood, it cannot be too strongly impressed that whereas their preliminary studies are merely a means of fitting them for their later ones, the latter differ entirely in being a direct preparation for their after-life and careers. It follows therefore that if there is one period more than another during which an effort should be made to secure a real proficiency, which shall be lasting and beyond examination requirements, that period is essentially the clinical one, when in the wards, operating theatres, casualty, rooms, and other subdivisions of a teaching hospital the student is laying the foundations of something far more valuable than degrees or diplomas, namely, clinical experience.

The conclusion is that during student years he is wise who, intending to practise medicine, or surgery, or midwifery, or all three, makes his preliminary studies strictly a means to the passing of the selected examinations and gets done with them as quickly as he can; on the other hand, he who adopts similar slapdash tactics with his final examinations may indeed, by luck or successful cramming, obtain a qualification to practise, but is yet doing a foolish thing and one he may ultimately have cause to regret. If, however, he proposes to take the appointment of a resident medical officer in a hospital, he has then the opportunity of his life for acquiring clinical wisdom, and a somewhat hurried preparatory student career may not stand in his way if he is ready and willing to learn and to cover the deficiencies of training by reading. To plan out beforehand, roughly and in outline, the ideal and hoped-for course of study and examinations is, therefore, a scheme which is worth the serious attention of those beginning medical study. Many things—for example, ill-health or private affairs, or idleness—may arise to cause wide departure from such an ideal; and those must be reckoned not only praiseworthy, but fortunate also, who keep to their schedule and meet with no check to their progress up the educational ladder. Yet if for each item in which the result falls short of the ideal a reason can be assigned; if the student on reaching his qualification can say where and why and how some of his time was wasted; then equally he is entitled to the satisfaction of knowing that otherwise and apart from such demonstrable causes of delay or of partial failure he has deserved his success by patiently seeking and toilfully ensuring it.

